# A Group-Based 8-Week Functional Interval-Type Outdoor Training Program Improves Physical Performance in Recreationally Active Adults

**DOI:** 10.3389/fspor.2021.627853

**Published:** 2021-03-31

**Authors:** Anna Hendker, Eric Eils

**Affiliations:** Department of Neuromotor Behavior and Exercise, Institute of Sport and Exercise Sciences, University of Münster, Münster, Germany

**Keywords:** functional training, circuit training, multimodal exercise program, high-intensity interval circuit training, group training, core stability

## Abstract

Even though physical activity is an important aspect of health, lack of time or motivation impede people from working out regularly. One type of training program that is both efficient and motivating is functional interval-type outdoor training. To evaluate this, our study had 81 participants complete a battery of physical performance tests (incremental treadmill test, core stability test and functional fitness test) before and after an 8-week outdoor interval-type training intervention. Training procedures included multimodal and high-intensity exercises performed in consistent, small groups. Results showed that the interval training program produced significant increases in the intervention group (*n* = 43) in functional and strength exercises (*p* < 0.001; squat (+27%), burpee (+24%), bridge (+39%), push-up (+34%), sit-up (+25%), high knees (+25%), row (+19%), effect sizes 0.4–0.11) for almost all parameters in comparison to a non-intervention control population (*n* = 38). Interestingly, trunk stability increased significantly (*p* < 0.001; flexion (+86%), extension (+43%), lateral left (+39%), lateral right (+32%), effect sizes 0.3–0.15) even though it was not explicitly trained; this was rather a secondary outcome of the functional exercises. Drop-out rates (Intervention group: 27%) suggest that this form of training seems to improve adherence to exercise and may help participants to stay committed to regular, intense activity. By simultaneously triggering adaptations in functional fitness, endurance and whole-body movements, this program can be used by people at different training levels.

## Introduction

Regular physical activity is indispensable for health (Blair et al., 1989; Brill et al., 2000; Haskell et al., 2007); however, worldwide, about 25% of adults do not adhere the WHO guidelines (World Health Organization, 2018). The most common reasons for sedentary behavior in the average population appear to be lack of time or motivation (Stutts, 2002; Trost et al., 2002; Korkiakangas et al., 2011). Since these reasons for inactivity may reflect individual personal traits, well-organized programs are needed to encourage people with limited spare time to participate. Thus, in contrast to more traditional time consuming low-intensity programs, many of the more time-efficient programs include short but intense training sessions. A combination of moderate- and vigorous-intensity activity enjoys increasing popularity in recent years (Batrakoulis, 2019; Thompson, 2021) and can be performed to meet the recommendation defined by the American College of Sports and Medicine (Haskell et al., 2007).

One such method is high-intensity interval training (HIIT) (Jones et al., 2011; Batrakoulis et al., 2020). By combining short, high-intensity bouts and e.g., functional strength exercises, this method aims for maximal training success with minimal time effort (Roy et al., 2018). The benefits of this type of training program have been shown repeatedly for various unhealthy and healthy populations (Nybo et al., 2010; Gibala et al., 2012; Batrakoulis et al., 2018, 2020). For patient groups, these programs are especially beneficial for improving peoples' motivation, body composition and functional movements (Nybo et al., 2010; Gibala et al., 2012; Midtgaard et al., 2013; Kampshoff et al., 2015; Devin et al., 2016; Schmitt et al., 2016). HIIT protocols offer superior overall adaptations (Gibala et al., 2012; Greenlee et al., 2017) and also lead to adjustments in cardiorespiratory fitness (VO_2_max) and aerobic performance (Buckley et al., 2015; Marterer et al., 2019). HIIT requires less exercise volume and demonstrating high compliance rates when conducted under supervision in untrained individuals (Roy et al., 2018). But, although HIIT has gained in popularity recently, these time-efficient but intense workouts include supramaximal efforts of exercise modes which might be difficult to reach in a group-based training session.

Another training method in which participants can realize a wide variety of athletic improvements is called functional or multimodal training (hereafter: *functional training*, FT). FT combines exercises that involve speed, power and strength, contains various resistance and body-weight modalities (Buckley et al., 2015) and uses a multidimensional approach, whereby exercises involve complex movement patterns and, thus, allow participants experience a relatively wide range of motion (e.g., single leg deadlift). This type of training can also include free weights and unilateral exercises, thus targeting many stabilizing muscles (Boyle, 2017), which may increase oxygen cost and yield functional and proprioceptive adaptations (Beckham and Earnest, 2000). Typical FT sessions include whole-body exercises using small equipment (e.g., kettlebells, battle ropes, etc.) and can be executed in interval and circuit weight training regimes (Skidmore et al., 2012).

Including free-weight exercises in a circuit format has been previously reported to produce many different gains in physical fitness (e.g., flexibility, muscle strength, muscle endurance, power, body composition, cardiovascular and cardiorespiratory endurance) in recreationally active females (McRae et al., 2012; Buckley et al., 2015; Myers et al., 2015) and healthy men (Schaun et al., 2018), in a relatively short amount of time. Acute adjustments were also detected, such as higher blood lactate concentrations, heart rate (HR) and rating of perceived exertion (RPE) values (McRae et al., 2012; Skidmore et al., 2012; Buckley et al., 2015; Sperlich et al., 2017). A continuous circuit-based whole-body aerobic resistance training program with rotated exercises in a fixed time span can elicit a greater cardiorespiratory response and similar muscular strength gains with less time commitment compared with a traditional resistance- and aerobic training program (Myers et al., 2015). But an interval- type regime with functional whole body movements like e.g., jumping jacks or burpees (McRae et al., 2012) has limited effects on strength. Therefore, the implementation of equipment into a functional interval training might be beneficial in order to reach more strength adaptations. Due to the complexity of these exercises and that they require a certain amount of coordination skills, guided training appears to be necessary to ensure that exercises are conducted in a healthy and safe manner.

Focusing on both interval circuit training and FT, a combination of these two modalities may help participants prepare for and meet everyday movement demands and athletic performance because FT methods offer range of motion increases and therefore potential improvements to stability. Further, combined trainings like these can easily be realized in a circuit format, e.g., outdoors. Working out in a natural environment is associated with additional benefits to those experienced after working out in an indoor environment (Pretty et al., 2003). Furthermore, it increases positive feelings (e.g., revitalization, positive engagement, decreases in tension) and leads to an increased likelihood of repeating the activity at a later date (Thompson Coon et al., 2011). As far as we know, knowledge about the effect of a similar training in an outdoor environment is limited and meriting a closer look.

Given the potential benefits of circuit training, FT and performing workouts outdoors, the aim of the present study was to evaluate a fitness training concept that combines all three elements. Since the benefits of these individual elements have been recently identified, combining them have shown to be a promising approach for people to improve their fitness in a time-efficient way. Previous investigations have shown promising, but also different outcomes depending on the specific training protocol (de Vreede et al., 2005; Buckley et al., 2015; Myers et al., 2015; Roy et al., 2018; Evangelista et al., 2019; Menz et al., 2019; Batrakoulis et al., 2020). We hypothesize that functional, and intensive interval training in a circuit format will lead to significant improvements in physical fitness in a training group compared to a non-intervention control group. Due to the combination of whole-body and (high-)intensity exercises, we expect a transfer effect, i.e., improvements will not exclusive occur to the test outcome of the functional exercise but also for isolated core and endurance exercises/ tasks that were not specifically trained.

## Methods

### Participants

Originally, 102 recreationally active participants from two cities in Germany were recruited via notice board, email distribution (by health insurance and companies) and personal networking. They were allocated into an intervention group (IG, *n* = 59) and an age-, activity- and gender-matched control group (CG, *n* = 43). Due to organizational reasons, participants were informed beforehand whether they would take part in the twice-weekly training sessions or whether they would belong to the CG. Those in the CG were asked to not change their typical activity level within these weeks. Data collection of the CG (pre- and post-test) was acquired 6 months after completion of the IG at similar weather conditions (late summer to autumn vs. late spring to early summer. Twenty-one percent of all participants dropped out, and 81 participants (IG: *n* = 43; CG: *n* = 38) were included in the analysis. Anthropometric data of all included participants (*n* = 81) is presented in Table 1.

**Table 1:** Anthropometric data (means and standard deviations) of included participants (*n* = 81).

	**Intervention group**			**Control group**			***P***
	**All**	**Female**	**Male**	**All**	**Female**	**Male**	
*n*	43	30	13	38	24	14	
Age (years)	33.9 ± 6.8	33.6 ± 7.0	34.5 ± 6.1	28.7 ± 6.2	27.5 ± 5.8	30.8 ± 6.2	0.001
Height (cm)	174.7 ± 8.8	170.6 ± 5.8	183.8 ± 7.5	175.7 ± 9.9	171.1 ± 8.0	183.8 ± 7.5	0.628
Weight (kg)	71.9 ± 12.3	66.6 ± 8.5	83.8 ± 11.0	72.7 ± 13.5	67.9 ± 11.1	80.7 ± 13.3	0.809
BMI (kg/m^2^)	23.5 ± 3.1	22.9 ± 3.0	24.8 ± 2.8	23.4 ± 3.3	23.2 ± 3.3	23.8 ± 3.2	0.915

Participants were determined to be “recreationally active” if they trained 2–8 h per week (e.g., jogging, fitness, cycling). Participants were excluded if they (1) reported any history of disease that limited their ability to perform the submaximal testing and the trainings, and, (2) had previously participated in a regular functional intensive training during the last 6 months. In addition, participants who failed to attend at least 80% of all training sessions were not included in the data analysis. All participants were healthy and were asked not to change their regular daily activities and dietary habits throughout the study duration.

**Figure 1:**
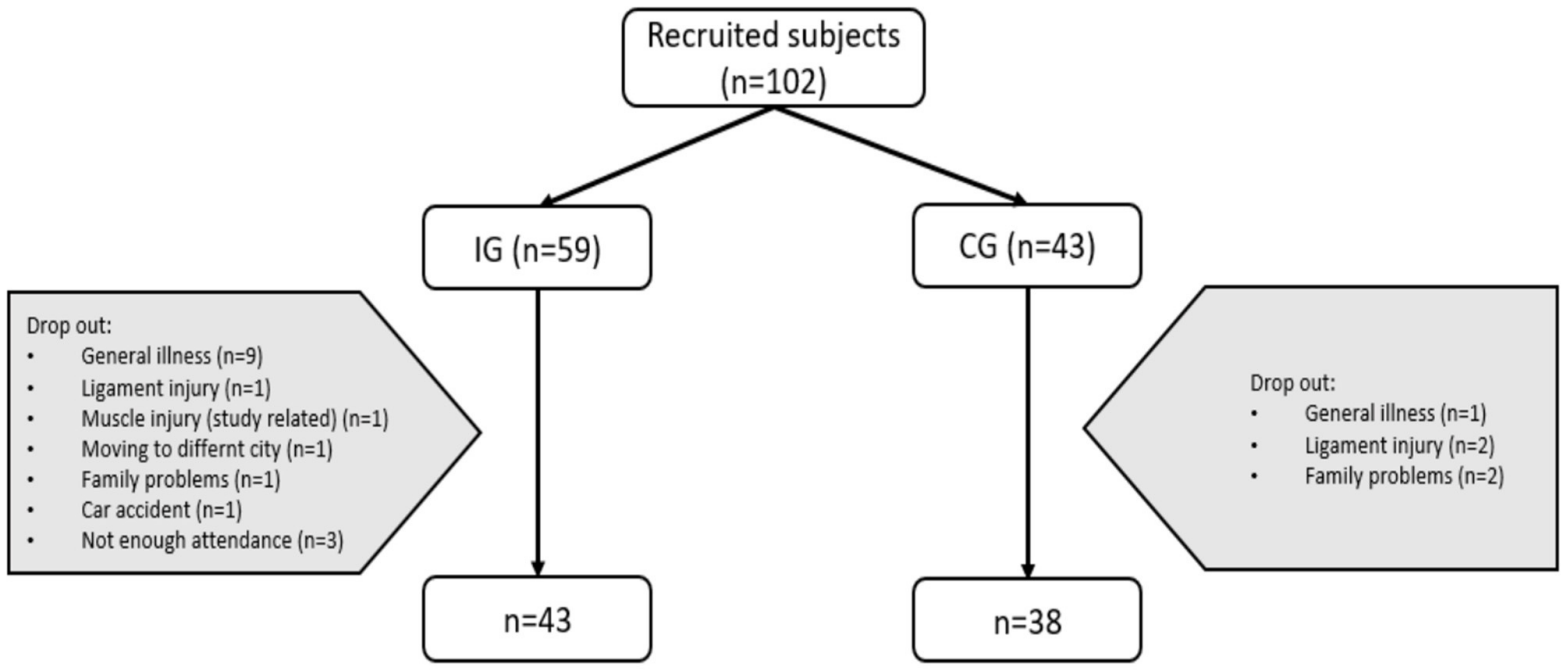
Flowchart of participant recruitment and reasons for drop-out.

Drop-out rates in the IG (27.1%) and CG (11.6%) were similar due to reasons including general illness or family problems (Figure 1). In the end, 81 participants completed the study (Table 1). The study was approved by the local Ethics Committee of the Department (2019-33-AH), and all participants were informed about the design and the procedure of the study and gave written consent for their participation according to the Declaration of Helsinki.

### Intervention

**Figure 2:**
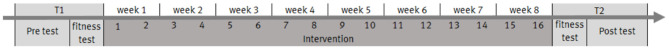
Temporal overview of intervention and test design.

Participants completed a battery of physical performance tests in a pre- and post-test design. During an 8-week outdoor intervention (Figure 2), they performed a guided 1 h functional outdoor circuit training in small groups (14 persons) twice a week with a qualified personal trainer. The type of training could be declared as “HIIT-type training” because it aims to raise HR to submaximal level twice per round (at least two out of seven exercises were especially cardio- demanding) but since the required HR-level are sometimes hard to reach in a group-session with own body movements, we label our training as “functional interval-type outdoor training.”

**Figure 3:**
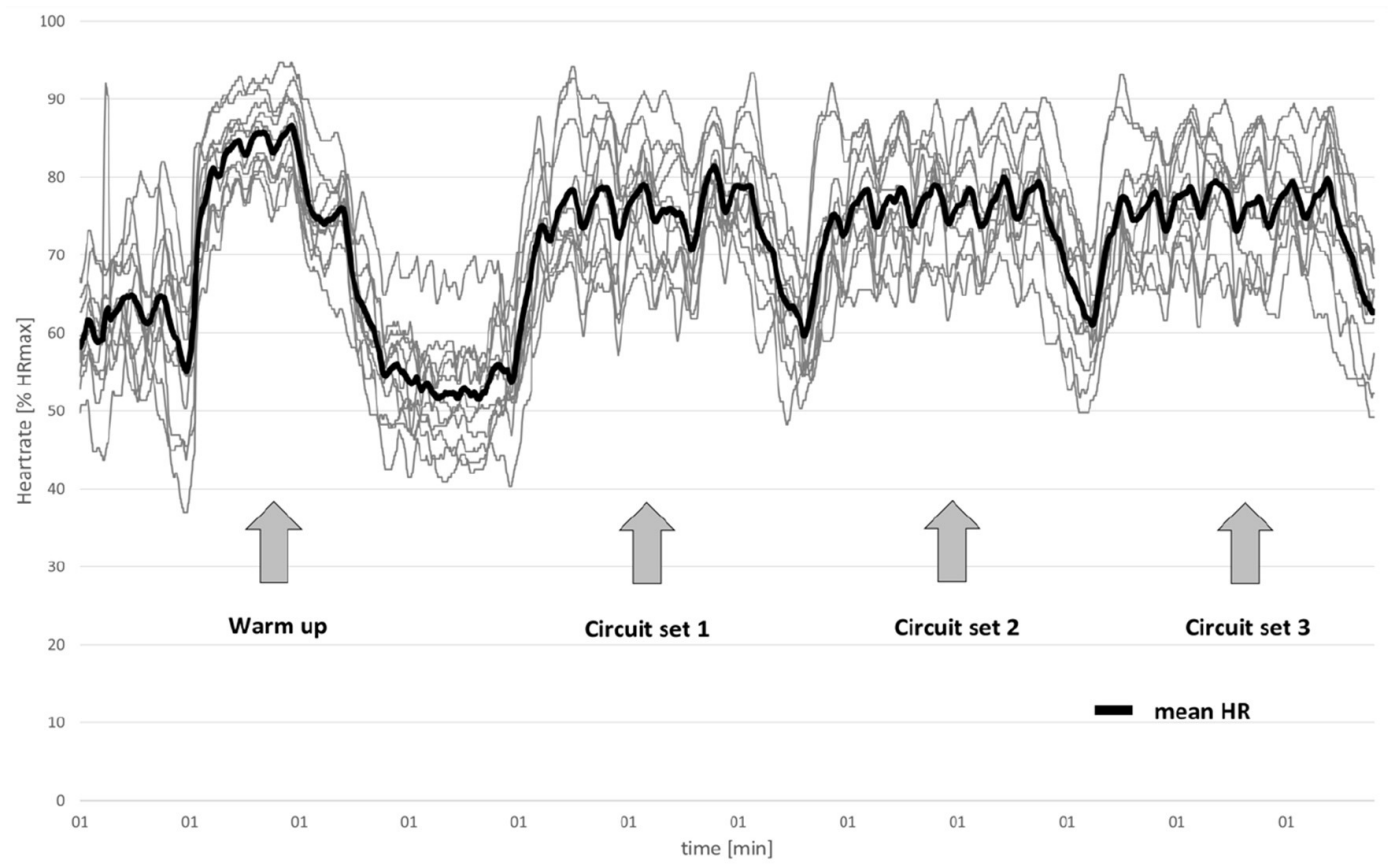
Example of mean (thick line) and single heart rates (thin lines) in one training session (*n* = 11).

The beginning of each session consisted of a 10 min warm-up including exercises to gradually increase range of motion and heart rate. Warm-up started from the feet to the head (slightly moving each joint through its range of motion), followed by larger and more complex movements (e.g., sidesteps, lunges, plank- or deep squat positions), small games (e.g., relay games) or little jumps (e.g., jumping jacks). This part was guided in a setting where participants were arranged in a circle and followed the instructions of the coach. Next, the instructor organized a circuit of seven different stations where one exercise should be performed for 1 min. The instructor demonstrated each of the seven exercises at three levels of difficulty; the participants chose one of these three level individually for each exercise (regression and progression) and were allowed to adapt the individual level to ensure proper load and strain. A burpee e.g., was a jump from plank to squat to upright jump and back again. The regression was e.g., the same movement but walking the feet instead of jumping and the progression was to include a push-up and to increase speed. Exercises were whole-body movements, some of which included small training equipment such as kettlebells or dumbbells to increase the load and to reach the defined conditions of multimodal or functional exercises, as well as to introduce variety. The seven exercises differed on each training day (for variety and motivation), but at least two of the seven exercises were aimed at cardiovascular demands so that participants reached HR values close to 80% of their maximum heart rate (HRmax). Each station lasted 60 s with a 15 s rest between stations; after the circuit of seven stations, there was a 2 min rest. A detailed exercise schedule is offered as Supplementary Material. Circuits were performed together with a self-chosen partner out of the group three times. To improve enjoyment and variation in the training program, three sessions offered other intense training protocols (e.g., one Tabata circuit where each of the seven stations was one tabata-training with two alternating exercises), and motivation was also kept up by intense verbal encouragement by the instructor (at least one per minute individually for each person out of the training group and overall for the team). In addition to offering motivational support, the experienced instructor ensured appropriate execution of the different exercises. Participants were instructed to complete as many repetitions as possible during this 1 min bout at every station. To check for training intensity objectively, the IG was observed via a group HR tracker (Acentas HMR Team, acentas GmbH, Hörgertshausen, Germany) twice in this 8-week period (Figure 3). RPE rates were documented once per week directly after training to evaluate subjective exhaustion. The control group underwent the same pre- and post-tests without performing the 8-week training period.

### Test Design

Physical performance of IG and CG were assessed in pre- and post-tests (1 week before starting the intervention and 1 week after finishing the last training) by the same test instructors, at the same time of day and in the same order. Assessments were a specific functional fitness test (OBC Fitness test, Original Bootcamp, Cologne), an incremental treadmill test with stepwise increasing speed, lactate and HR analysis, and a core stability test (McGill, 2007) for transferred performance adjustments.

After arriving at the laboratory, participants were informed about the study, the measurement procedures and the intervention. They had time to ask questions and to get familiar with the situation and the instructors. After giving their written informed consent, their weight, height and personal data were assessed. They filled out the questionnaires and received data privacy protection information. Afterwards, blood lactate (BLa) was collected at rest in 20 μl samples of capillary blood, withdrawn from the earlobe, and was subsequently measured using an enzymatic method. The core stability test followed under standardized rest times and after a short break, BLa was collected prior to the incremental treadmill test, after every step, and again immediately after completion of the test as well as at 5 and 10 min after completion of the test.

#### Core Stability

The McGill core stability test (McGill et al., 1999; McGill, 2007) assesses whole-body stability by evaluating core muscle endurance capacity for prevention purposes. Participants needed to remain in defined and controlled positions for the torso flexors, torso extensors and lateral musculature until maximal voluntary exhaustion. The order of the four test positions followed a random principle of chance, but was constant in pre- and post-test for each participant. Rest time between each position was 2 min. Test instructors measured the time they held these positions correctly; generally, the ratio of the time that different positions are held can indicate back problems (McGill et al., 2003). Although the instructors were well-educated and qualified to judge the correct outcome of the positions, we made sure that the same person was assigned for pre- and post-test measurement.

#### Incremental Treadmill Test

After a short rest, the incremental treadmill test was completed. This was performed in 3 min steps (DeMarées, 2017) on a treadmill (h/p/cosmos pulsar® 3p; No. cos30004va04) and conducted under controlled conditions to assess maximum running distance (dist), HR, maximum speed (msp), lactate accumulation (BLa), rating of perceived exertion (RPE), and time (t). Participants started walking on the treadmill at a speed of 4 km/h for 3 min, followed by a 30 s rest that was used to take blood samples and to note the HR. The next step started at a speed of +2 km/h. This procedure was continued until maximum voluntary exhaustion; namely, the participants ran until their maximum exhaustion and were verbally encouraged by the staff. After test termination, the participants rated the exertion on the Borg Scale ranging from 6 to 20 (Skidmore et al., 2012). Lactate samples and HR were also noted directly at the end of the test and at 5 and 10 min after test completion (during recovery). After this test, the participants finished the pre-test measurements. The tests were performed in the same order in the post-test measurements 1 week after the last training session.

#### Functional Fitness Test

One week later, after a standardized warm-up and familiarization with the exercises and the other participants, the functional fitness test was performed outside. To evaluate maximal repetitions, a proper surface condition was secured to warrant sufficient grip. The functional fitness test included seven exercises typical of whole-body fitness training: squats, burpees, hip bridges, push-ups, sit-ups, high knees and rowing. The order of these exercises was fixed, but each participant choose one starting point which was constant for pre- and post-test. Participants started at one of these seven stations and performed the exercise for 1 min. Depending on the level of difficulty, additional weight was used via kettlebells and dumbbells. For example, there were four different level of squats, two with additional weight: squat with elevated heels on a rolled gymnastics mat (level 1), squat with elevated arms (“Y-squat”-level 2), squat with a 8 kg Kettlebell (pressed on the chest-level 3), squat with a 12 kg Kettlebell (level 4). The depth of the squat was normalized by means of an external object fixed on height of the hollow of the knee. Furthermore, exercises were leveled by technical parameters, e.g., three level of burpees. Exercise order and level were the same in the pre- and post-tests for each participant. Every exercise, with its progressions and regressions, was explained and demonstrated by the same instructor. Maximal repetitions and the individual level were documented.

### Statistical Analysis

For all data, means and standard deviations (SD) were calculated. A mixed ANOVA with one within-subject factor (time: pre- and post-test) and one between-subject factor (group: intervention and control) was calculated. Effect sizes were described with partial eta squares. The alpha level was set to 5%. In case of significant interactions, the type of interaction was classified into ordinal, disordinal or semi-disordinal interactions (Döring and Bortz, 2016) to ensure the interpretability of the main effects. For ordinal interactions both main effects were interpreted; for semi-ordinal interactions only one factor (time or group) was interpreted; and for disordinal interactions none of the main effects were interpreted. The differences between pre and post-test for each group were analyzed separately as a *post-hoc*-test (paired *t*-test) when significant interactions occurred. All statistical tests were carried out using SPSS Statistics software (version 25, SPSS Inc., Chicago, IL, USA).

## Results

All participants of the IG who completed at least 14 of the 16 offered training sessions were included in the analysis (*n* = 43). There were no significant differences between the groups at baseline. RPE in training indicated that the training was “hard” with a mean of 15.9 ± 0.9 points on the Borg Scale and an HR_max_ of 190 beats per minute. HR was tracked in two sessions per training group to evaluate the intensity. Mean HR values of a typical training session (1 h) including the warm-up and three circuit sets of seven exercises each (145.6 ± 21.9 bpm, 147.4 ± 19.7 bpm and 148.22 ± 18.89 bpm in first, second and third circuits, respectively) indicated moderate to high intensity (Figure 3).

The results of the ANOVA revealed significant interactions (ordinal, semi-ordinal and disordinal) between the factors *time* and *group* for almost all parameters (Table 2). Thus, a simple presentation and discussion of their main effects on dependent variables was not directly possible without analyzing the type of interaction.

**Table 2:** Mean values, standard deviations (SD) and results of ANOVA of evaluated parameters for IG and CG during pre- and post-tests.

		**Intervention group**		**Control group**									
		**Pre-test**	**Post-test**	**Pre-test**	**Post-test**	**ME (time)**			**ME (group)**			**IE (group x time)**		
	**Parameter**	**Mean ± SD**	**Mean ± SD**	**Mean ± SD**	**Mean ± SD**	***F*-value**	**p level**	**pEta^**2**^**	***F-*value**	**p level**	**pEta^**2**^**	***F*-value**	**p level**	**pEta^**2**^**
Incremental step test	Distance [m]	2227.8 ± 601.7	2491.4 ± 742.4	2663.2 ± 722.2	2491.3 ± 754.9	(1, 76)= 1.74	=0.191	0.02	(1, 76)= 1.94	=0.168	0.030	(1, 76)= 39.36	**<0.001**	0.34
	Speed at 4 mmol/l lactate [km/h]	10.1 ± 1.9	10.6 ± 1.9	11.2 ± 1.9	11.1± 1.9	(1, 76)= 3.15	=0.080	0.04	(1, 76)= 3.63	=0.061	0.050	(1, 76)= 6.42	**=0.013**	0.08
	HR at 4 mmol/l lactate [bpm]	169.7 ± 12.6	168.3 ± 10.5	173.7 ± 12.6	173.0 ± 13.1	(1, 76)= 1.26	=0.266	0.018	(1, 76)= 2.56	=0.114	0.036	(1, 76)= 0.156	=0.694	0.002
	RPE [0–20]	17.2 ± 1.4	18.3 ± 1.4	17.7 ± 1.2	17.5 ± 1.3	(1, 76)= 7.97	**=0.006**	0.095	(1, 76)= 0.52	=0.475	0.007	(1, 76)= 19.12	**<0.001**	0.201
Core stability test	Flexion [sec]	48.8 ± 22.3	90.8 ± 39.8	69.2 ±24.2	67.8 ± 26.6	(1, 76)= 28.67	**<0.001**	0.271	(1, 76)= 0.06	=0.815	0.001	(1, 76)= 32.73	**<0.001**	0.298
	Extension [sec]	78.3 ± 30.3	111.7 ± 41.0	92.7 ± 40.0	87.1 ± 47.4	(1, 76)= 13.92	**<0.001**	0.151	(1, 76)= 0.39	=0.532	0.005	(1, 76)= 27.46	**<0.001**	0.260
	Lateral left [sec]	57.2 ± 23.2	79.3 ± 29.9	67.5 ± 29.3	64.7 ± 29.9	(1, 76)= 19.76	**<0.001**	0.202	(1, 76)= 32.64	=0.723	0.002	(1, 76)= 0.127	**<0.001**	0.295
	Lateral right [sec]	59.9 ± 28.4	78.8 ± 32.8	68.2 ± 30.8	69.3 ± 32.3	(1, 76)= 17.24	**<0.001**	0.181	(1, 76)= 0.01	=0.923	0.000	(1, 76)= 13.81	**<0.001**	0.150
Functional fitness test	Squat [RPM]	27.8 ± 5.6	35.2 ± 5.9	35.3 ± 8.4	34.7 ±7.8	(1, 76)= 38.23	**<0.001**	0.335	(1, 76)= 5.74	=0.019	0.070	(1, 76)= 51.36	**<0.001**	0.403
	Burpee [RPM]	16.4 ± 4.0	20.3 ± 3.1	20.1 ± 4.7	20.4 ± 4.9	(1, 76)= 32.37	**<0.001**	0.299	(1, 76)= 4.52	=0.037	0.056	(1, 76)= 32.37	**<0.001**	0.299
	Bridge [RPM]	45.4 ± 11.4	63.3 ± 11.5	60.1 ± 15.1	59.9 ± 14.4	(1, 76)= 40.46	**<0.001**	0.347	(1, 76)= 4.65	=0.034	0.058	(1, 76)= 42.58	**<0.001**	0.359
	Pushup [RPM]	19.7 ± 7.2	26.4 ± 8.7	20.8 ± 10.5	20.9 ± 10.4	(1, 76)= 43.94	**<0.001**	0.369	(1, 76)= 1.15	=0.288	0.015	(1, 76)= 41.67	**<0.001**	0.357
	Sit up [RPM]	19.2 ± 6.7	24.0 ± 6.0	20.6 ± 7.3	21.6 ± 7.3	(1, 76)= 45.17	**<0.001**	0.373	(1, 76)= 0.12	=0.726	0.002	(1, 76)= 19.11	**<0.001**	0.201
	High knees [RPM]	136.1 ± 29.4	170.4 ± 32.8	160.4 ± 23.9	158.5 ± 28.6	(1, 76)= 36.81	**<0.001**	0.326	(1, 76)= 1.06	=0.306	0.014	(1, 76)= 46.19	**<0.001**	0.378
	Row [RPM]	22.1 ± 4.6	26.2 ± 5.0	24.7 ± 7.9	25.3 ± 7.8	(1, 76)= 18.65	**<0.001**	0.197	(1, 76)= 0.40	=0.529	0.005	(1, 76)= 9.68	**=0.003**	0.113

*Statistics are presented for the main effects (ME) time and group and the interaction effect (IE) time × group. Bold values indicate significant differences for main effects and/or interventions*.

Analysis of the *incremental* treadmill *test* showed significant interactions concerning distance, speed at 4 mmol/l lactate, and RPE. RPE also showed a significant main effect of time that was not interpretable due to a disordinal interaction. No significant main effect or interaction was found for HR at 4 mmol/l lactate (Table 2). Effect sizes ranged between 0.002 (HR at 4 mmol/l lactate) and 0.34 (distance). Significant interactions revealed that the IG improved performance for distance, speed at 4 mmol/l lactate and RPE, whereas the CG's performance remained or declined. *Post-hoc* analysis showed that performance significantly increased in the IG for distance (*p* <0.001), speed at 4 mmol/l lactate (*p* < 0.01), and RPE (*p* < 0.001). The CG showed a significant decrease in performance for distance (*p* < 0.01) and no differences for speed at 4 mmol/l lactate (*p* = 0.544), and RPE (*p* = 0.270). The distances covered from IG and CG were comparable in the post-test but different in the pre-test, indicating that the CG started from a higher performance level.

*Core stability* analysis revealed significant interactions for all parameters (flexion, extension, lateral right and lateral left) and significant main effects for time but not for group (Table 2). Interactions for flexion, extension, and lateral right were disordinal (main effects not interpretable) and semi-ordinal for lateral left (main effect time, interpretable) (Table 2). Effect sizes for all parameters ranged from 0.30 (flexion) to 0.15 (lateral left). Significant interactions revealed that the IG showed improved performance for flexion, extension, lateral right and lateral left, whereas the CG's performance remained or declined. *Post-hoc* analysis showed that performance significantly increased in the IG for flexion (*p* < 0.001), extension (*p* < 0.001), lateral right (*p* < 0.001), and lateral left (*p* < 0.001). The CG showed no significant differences in performance for flexion (*p* = 0.780), extension (*p* = 0.304), lateral right (*p* = 0.381), and lateral left (*p* = 0.774).

The ratios between positions (Table 3) could be compared with reference values from the literature (McGill, 2007); for a healthy core, it is desirable to attain a ratio close to the reference ratios. After the intervention, the flexion/extension ratio did not change. Yet, ratios between sides to extension improved in IG only, as did the right to left side ratio, which better approximated a recommended level in the IG compared to in the CG.

**Table 3:** Ratios of different core exercises before and after the intervention ± standard deviation.

	**Intervention group**		**Control group**		
	**Pre-test**	**Post-test**	**Pre-test**	**Post-test**	**Reference**
Flexion/Extension	0.69 ± 0.33	0.88 ± 0.41	0.87 ± 0.42	0.94 ± 0.51	<1.00
Lateral R/Extension	0.82 ± 0.44	0.75 ± 0.23	0.81 ± 0.34	0.82 ± 0.29	<0.75
Lateral L/Extension	0.89 ± 0.58	0.75 ± 0.25	0.80 ± 0.29	0.89 ± 0.40	<0.75
Lateral R/Lateral L	1.05 ± 0.39	1.03 ± 0.16	1.01 ± 0.24	0.96 ± 0.21	0.95–1.05

The *functional fitness test* results showed significant interactions for all parameters (squat, burpee, bridge, push-up, sit-up, high knees, row) regarding the number of repetitions per minute, significant main effects for time, and significant main effects for the factor group concerning the parameters squat, burpee and bridge (Table 2). Interactions for squat, bridge, and high knees were disordinal (main effects for time and group not interpretable), for burpee were ordinal (both main effects time and group are fully interpretable), and for push-up, sit-up and row were semi-ordinal (main effect of time is fully interpretable) (Table 2). Effect sizes ranged between 0.40 and 0.11 for all parameters. Significant interactions revealed that the IG showed improved performance for all functional fitness exercises, whereas the CG's performance was nearly unaffected. *Post-hoc* analysis showed that performance significantly increased in the IG for squat (*p* < 0.001), burpee (*p* < 0.001), bridge (*p* < 0.001), push-up (*p* < 0.001), sit-up (*p* < 0.001), high knees (*p* < 0.001), and row (*p* < 0.001). The CG showed no significant differences in performance for squat (*p* = 0.4911), burpee (*p* = 0.544), bridge (*p* = 0.921), push-up (*p* = 0.917), sit-up (*p* = 0.119), high knees (*p* = 0.652), and row (*p* = 0.506).

## Discussion

The aim of the present study was to evaluate the effects of an outdoor circuit training concept on recreationally active participants without specific prior experience (or at least in the recent 6 months) in functional training. To monitor possible changes in participants' fitness status, we assessed cardiorespiratory fitness, core stability, and functional fitness skills. The results demonstrate that many parameters concerning functional fitness and core stability improved due to the training intervention. Enhancements in functional fitness exercises were expected, but improvements were also found in tasks and exercises that were not trained specifically within this training program (e.g., endurance performance). Interestingly, all parameters of the incremental treadmill test also increased significantly, except HR at 4 mmol/l lactate, thus underlining the transferability and efficacy of such a training program.

Despite the relatively challenging task requirement (participants had to join at least 14 out of 16 offered training sessions on fixed days over an 8-week period and offer four appointments for pre- and post-testing), 27.1% of the IG dropped out. Reasons are not only the missed attendance, but also illness, injuries and family problems (Figure 1). This aspect suggests that participants showed high compliance and were motivated to engage in a regular and time-efficient activity with this concept. Further, almost one quarter of the participants reported in the post-test that they will continue this type of training after finishing the study, which indicates that the concept successfully motivated people to perform in regular intensive activity. These findings were in line with other reported results (Oliveira et al., 2018; Batrakoulis et al., 2020). Participants' motivation is often increased in courses with a fixed social framework, as these courses try to combine the training with the joy of social interaction. Moreover, motivation (or at least participation) may have been enhanced by the constant presence of a personal trainer and a consistent peer group (maximum 14 participants in one training group); these factors may have led to strengthened social cohesion and attendance. An additionally motivator could have been these classes were conducted outdoors, as training in the fresh air and in nature results in greater feelings of revitalization and numerous other psychological enhancements compared to indoor training (Thompson Coon et al., 2011). Overall, the combination of fixed groupmates, an instructor that supports and encourages participants, fixed 1 h sessions twice per week, and exercising outdoors in nature seems to keep participants committed to regular intense workouts, which improved their physical fitness over 8-weeks.

### Implications on Core Stability

Through the training, IG participants improved not only in performing specific exercises, but they also improved their general core stability. This finding is in line with our hypothesis. Isolated core exercises like the tested positions were not part of the weekly training cycle, which means that the training resulted in a transfer effect to, for example, trunk stabilizers. The applied multimodal whole-body exercises in this study strengthened the stabilizing trunk muscles and may have a positive effect for everyday life. A strong core is additionally helpful for many other athletic tasks, such as changing directions or being able to withstand external impacts (Hibbs et al., 2008). Moreover, functional resistance training improves (functional) strength (Sperlich et al., 2017, 2018; Cortell-Tormo et al., 2018).

In our study, even when the effect sizes were low, participants improved performance substantially in core strength endurance with retention times of ≥75 s. Comparing the ratios between the positions in the intervention group with reference values from the literature (McGill, 2007), we see a clear preventive effect of the training in terms of potential back pain (Table 3). This outcome is in line with previous findings showing that functional exercises trained via a tabata protocol resulted in increased trunk strength (Engel et al., 2019). Therefore, the applied training method could be recommended as a prevention program due to its beneficial effects on core stability.

### Implications on Endurance Performance

The training effect on endurance capabilities reveals interesting outcomes. Running distance and speed at 4 mmol/l lactate showed significant interaction effects, which can be attributed to a performance increase in the IG. However, focusing on the mean values of distance and speed, participants in the CG showed a higher pre-test value than the IG participants had in the post-test. The CG ran longer in the incremental treadmill test prior to the intervention phase, and similar distances were reached in the post-test. For running velocity, the CG also had higher pre-test values than the IG in the post-test. This indicates that the significant interaction in these two cases must be interpreted with care. Furthermore, effect sizes were small for most parameter here. However, the IG increased their performance in all parameters of the incremental treadmill test, which might indicate that the training allowed them to develop a more economic metabolic process. This finding is in line with other studies where cardiovascular fitness, daily caloric expenditure and body composition was improved by similar trainings (Buckley et al., 2015; Vandenbrink et al., 2018; Menz et al., 2019). Yet, other studies have reported no improvement in running performance after a similar training (Engel et al., 2019).

In our study, the metabolic change was determined via lactate analysis. We focused on fixed lactate thresholds at 4 mmol/l lactate and calculated the current speed when reaching this limit. A combined circuit interval training produced higher blood lactate accumulation and RPE compared to traditional circuit weight training workouts or aerobic circuit training (Skidmore et al., 2012). While previous studies with similar training modalities showed positive effects on metabolism, our findings indicate a lower lactate accumulation in submaximal running velocity. Focusing on the HR, our findings at the 4 mmol/l threshold seem to contradict more pronounced reported decreased HR at a fixed 1.67 meter per second (m/s) velocity after a 4-week app-based HIIT session (Sperlich et al., 2018). However, our findings are in line with unaffected HR values at 1.67 m/s after a 9-week intervention with 2- to 3-weekly circuit HIIT sessions (Sperlich et al., 2017). These discrepancies might be explained by differences in calculating velocities at given lactate thresholds. Nevertheless, training protocols like ours aim at improving multimodal or functional strength by addressing large muscle groups in combination with bouts of increased HR close to the maximum; therefore, such protocols might not target the cardio-metabolic system enough to increase endurance-specific parameters. This is especially interesting because two cardio-focused training elements were included in every training in the intervention. After all, the term “HIIT” might not fit best in this context.

Focusing on RPE, results indicate that both groups were exhausted similarly in the pre-test, but the IG reported higher RPE values in the post-test. The likeliest explanation is that participants ran longer and felt more exhausted, indicating a better training status and/or being more familiar with experiencing maximal loadings. This is in line with other studies reporting outcomes after similar training regimes (Sperlich et al., 2017; Batrakoulis et al., 2018). On the other hand, we must consider that other factors could have influenced this outcome, e.g., participants in the IG spent more time with an instructor and might feel more obligated to give their full effort compared to the CG.

Collectively, results of the incremental treadmill test might be interpreted as the training potentially having a positive effect upon endurance capabilities even though no specific endurance (running) training was carried out.

### Implications on Functional Fitness

Our findings suggest that the IG significantly improved functional fitness performance across all seven exercises between pre- and post-tests compared to the CG, supporting our main hypothesis that the training intervention would lead to a significant improvement in physical fitness. These results are in line with recent results indicating that dynamic exercises in combination with training at high intensities can increase trunk muscle strength (Engel et al., 2019). The seven exercises we assessed (squat, burpee, bridge, push-up, sit-up, high knees, row) test different dimensions of body movement and comply with demands for multimodal or functional movements to meet the requirements of “everyday challenges.” These exercises were completed with the same, clearly defined level of difficulty in the pre- and post-test, and participants' increased number of repetitions indicated that they improved in performing multi-dimensional movements due to better intra- and intermuscular coordination (neuromuscular adaptation) or due to increased strength or endurance (morphologic or metabolic adaptation). It seems safe to assume that muscle coordination was not the reason for improvement, as the sessions consisted of different exercises, but the constantly changing movement pattern in the functional movement tasks may have enhanced movement variability, task adaptation and the ability to increase repetitions in the post-test. Due to a lack of suitable self-created or customized tests to evaluate functional fitness (McRae et al., 2012; Sperlich et al., 2017), we used a different self-generated test that has clearly defined movement qualities and levels of difficulties, both of which are supervised by well-trained staff members.

### Training Program

Two of the seven exercises in every circuit training within the training session were focused on cardiovascular fitness and required special motivation to ensure proper effects. RPE values after the training sessions indicate the sessions were “hard,” and they appeared to elevate HRs to >80% of HR_max_. The motivation provided by the instruction seemed especially important for pushing participants to their limits achieving increases in HR to about >80% of HR_max_. The training sessions demonstrated a moderate to high HR of 147 bpm, whereas the HR_max_ was at 190 bpm for all recorded sessions. Obviously, individual characteristics bias this result, but the relatively high HR mean over a 60 min period indicates a demanding training load (depending on individual HR_max_), since the average value included the warm-up phase (joint-by-joint mobilization) and the time during which the circuit was explained.

## Limitations

This study has some limitations that could impact the findings. The allocation of participants to the CG or IG was randomized after recruitment, but due to organizational reasons, members in the IG and CG were measured consecutively (members of the CG were measured after finishing the data acquisition of the IG). Moreover, although the randomization procedure to achieve group comparability focused on height, weight and level of physical activity, the drop-out rates changed this balance: The CG was slightly younger and in superior physiological condition compared to the IG. Thus, one question is whether the slightly worse condition of the IG allowed for greater improvement in this group; yet, the IG's higher mean age could have impeded it. Another issue is that we calculated the running velocity at the fixed 4 mmol/l threshold, dampening the individual nature of lactate expression and tolerance. Future research should measure individual changes in aerobic/anaerobic thresholds when assessing metabolic changes following this training modality. In comparison to previous research, shorter and more time-efficient training regimes (e.g., Tabata) are available in the current literature base, with similar beneficial outcomes (Skidmore et al., 2012; Smith-Ryan et al., 2016; Sperlich et al., 2018; Engel et al., 2019), but training modalities like ours offer different exercises to increase variation, provide a multi-joint warm-up to prevent injuries, and specifically prepare participants for the upcoming exercises. Altogether, within our investigation, we were able to evaluate a useful and time-efficient training program with a high number of participants by assessing the overall effect on different performance and prevention parameters like endurance capacity, core stability and multimodal fitness.

## Conclusion

A combination of functional and high-intensity exercises carried out in fixed, small groups significantly increased performance in comparison to a non-intervention control group. Participants not only improved at performing certain exercises, but they also improved in core stability and measures of endurance. By simultaneously triggering adaptations in strength, endurance and whole-body movement skills, this approach can be used as a diversified training method for people at different training levels. The combination of whole-body movements, in conjunction with an outdoor setting, a stable training group and a trainer led to moderate drop-out rates and appeared to increase motivation, leading to key physical enhancements that may benefit daily life activities. The special framework of the program (including equipment, training outside, a stable training group, and a trainer) may enhance participants' motivation and thus increase regular physical activity, which is indispensable for health. Due to these positive findings, future investigations should focus on the relevant health-promoting outcomes for specific target groups, e.g., age groups, highly active persons.

## Data Availability Statement

The raw data supporting the conclusions of this article will be made available by the authors, without undue reservation.

## Ethics Statement

The studies involving human participants were reviewed and approved by Ethics Committee of the Department of Psychology and Sport Sciences (University of Muenster) Number: 2019-33-AH. The patients/participants provided their written informed consent to participate in this study.

## Author Contributions

AH and EE: conceptualization, data curation, formal analysis, methodology, supervision, writing—original draft, and writing—review and editing. AH: funding acquisition, investigation, and project administration. EE: resources.

## Conflict of Interest

AH is an employee of OBC Europe GmbH and works as a trainer besides her normal job as a Ph.D student at the university. They offered help in recruiting participants for this study by using their newsletter for informing about the study. The work inspired me to conduct the study. The remaining author declares that the research was conducted in the absence of any commercial or financial relationships that could be construed as a potential conflict of interest.
